# Explaining Racial/Ethnic Disparities in Telehealth Use with Different Levels of English Proficiency: A Decomposition Approach

**DOI:** 10.1177/26924366251379188

**Published:** 2025-09-29

**Authors:** Nari Yoo, Yumin Hong, Yoonyoung Choi

**Affiliations:** ^1^ School of Social Work, University of Michigan, Ann Arbor, Michigan, USA.; ^2^ Department of Economics, University of Texas at Austin, Austin, Texas, USA.; ^3^ School of Sociology, The University of Arizona, Tucson, Arizona, USA.

**Keywords:** telehealth, telemental health, health disparities, service use, limited English proficiency, decomposition

## Abstract

**Background:**

This study examined disparities in telehealth utilization across various health care services among adults in California, with particular focus on racial, ethnic, and linguistic differences. While telehealth has emerged as a potential tool for addressing health care disparities, evidence suggests that utilization patterns may exacerbate existing inequities.

**Methods:**

Using the 2021–2022 California Health Interview Survey (n = 45,893) conducted in diverse languages, we employed descriptive statistics and nonlinear Blinder–Oaxaca decomposition to analyze disparities in telehealth utilization. The Blinder–Oaxaca method systematically partitions outcome differences between groups into explained components and unexplained components. We stratified analyses by visit purpose: mental health, primary care, acute care, chronic care, orthopedics, dermatology, and ophthalmology.

**Results:**

Compared with non-Hispanic Whites, African Americans used telehealth 4.1% more (*p* < 0.01), whereas Hispanics/Latinos and Asians used it 5.9% (*p* < 0.01) and 11.3% (*p* < 0.01) less, respectively. For Hispanics/Latinos, approximately 14% of the disparity remained unexplained by measured covariates, while for Asians, 92% was unexplained (*p* < 0.01). Individuals with limited English proficiency (LEP) were 9.6% (*p* < 0.01) less likely to use telehealth overall, with 38% of this gap unexplained by measured factors (*p* < 0.01). The disparity was most pronounced in mental health services, where LEP individuals utilized telehealth 4.9% points less than non-LEP individuals (*p* < 0.01), with this gap entirely unexplained by sociodemographic factors. Subgroup analysis revealed telehealth utilization disparities between LEP and non-LEP individuals across all language groups, with LEP Spanish speakers using telehealth 4.3% points less than non-LEP Spanish speakers (*p* < 0.01), LEP Asian language speakers using it 7.3% points less than non-LEP Asian language speakers (*p* < 0.01), and LEP speakers of other languages using it 7.3% points less than their non-LEP counterparts (*p* < 0.01).

**Discussion:**

Our findings reveal significant disparities in telehealth utilization associated with race/ethnicity and English proficiency levels, with linguistic barriers most evident in mental health services. The substantial unexplained components in our decomposition analyses suggest that cultural, structural, and linguistic factors beyond socioeconomic factors significantly influence telehealth utilization. These results underscore the need for efforts to develop linguistically appropriate telehealth services, particularly in mental health care. Addressing these barriers is crucial for harnessing telehealth’s potential to promote health equity rather than exacerbating existing health disparities in an increasingly digital health care environment.

## Introduction 

Telehealth has fundamentally transformed health care delivery, offering a promising approach to expanding care access, particularly for populations facing barriers to traditional in-person services.^1^ This transformation accelerated significantly during the COVID-19 pandemic, which necessitated rapid and widespread telehealth adoption across the United States.^2,3^ As a remote care modality, telehealth has emerged as a potentially powerful tool for addressing health care disparities among underserved populations.^4^ However, despite its theoretical potential to mitigate barriers in health care access, evidence suggests that telehealth utilization patterns exhibit significant racial/ethnic disparities.^5,6^

### Racial and ethnic disparities in telehealth utilization

While telehealth expansion has the potential to improve health care access, evidence suggests it may inadvertently exacerbate existing racial and ethnic disparities in health care.^7^ Several structural factors contribute to these disparities. Limited access to technology and broadband internet disproportionately affects low-income and rural areas with larger Black and American Indian/Alaska Native populations.^8^ Hispanic and Latino populations have shown a preference for in-person care, emphasizing culturally and linguistically appropriate telehealth services.^9^ In addition, lower digital literacy among older adults in minority groups also plays a role in racial and ethnic disparities in use.^10^ The digital divide—disparities in access to and use of information and communication technologies—further compounds access barriers for racial and ethnic minorities.^11,12^ Moreover, telehealth effectiveness and patient satisfaction vary substantially across different care needs, influencing utilization patterns.^13,14^

Research consistently demonstrates disparate telehealth utilization patterns across racial and ethnic groups. African Americans have generally shown lower rates of telehealth use compared to non-Hispanic Whites. In one large health care system study, Black patients were 40% less likely than White patients to access telehealth care, even after adjusting for socioeconomic factors.^15^ Similarly, among Medicare beneficiaries, Blacks had 29% lower odds of having a telehealth visit than Whites.^16^ However, some evidence suggests that when digital access barriers are controlled for, African Americans may demonstrate higher telehealth engagement for primary care.^16^ This is further supported by a study of a telehealth-delivered diabetes prevention program, which found high engagement and clinical benefits among African American participants.^17^

Hispanic and Latino populations consistently demonstrate lower telehealth utilization rates compared with non-Hispanic/Latino Whites. In a study examining over 200,000 patients, Hispanic and Latino individuals had significantly lower odds of telephone or video visits.^18^ Among Medicare beneficiaries, Hispanic or Latino individuals were 35% less likely than Whites to use telehealth.^16^ Research on Asian American telehealth utilization is more limited, but available evidence points to persistent disparities. Studies have found that Asian patients demonstrate lower odds of telehealth utilization compared with White patients^19,20^ and report lower satisfaction with telehealth communication, as observed among Asian breast cancer patients.^21^ One factor that may contribute to these disparities is language: 28% of the Hispanic and 31% of the Asian populations have limited English proficiency (LEP).^22^ These findings underscore the importance of examining how language proficiency influences telehealth utilization patterns.

### Linguistic disparities in telehealth use

While substantial research has examined racial and ethnic disparities in telehealth, linguistic barriers have received comparatively less scholarly attention. Emerging evidence, however, consistently demonstrates significant gaps in telehealth utilization between patients with limited English proficiency and without LEP. An analysis of California Health Interview Survey (CHIS) data found that patients with LEP had approximately half the odds of using telehealth compared with proficient English speakers, even after adjusting for other sociodemographic factors.^23^ Similarly, an examination of over 22,000 primary care visits scheduled by LEP patients found that only 34.5% were conducted via video, with substantial variation across language groups.^24^ Blundell^25^ further documented pronounced disparities in telemedicine access among Spanish-speaking patients, who demonstrated lower rates of scheduled appointments, email documentation, and patient portal activation compared to their English-speaking counterparts.

Several studies have investigated the factors contributing to reduced telehealth use among LEP populations.^23,26^ A significant barrier identified in the literature is the lack of language accessibility in telehealth platforms and services, since many telehealth systems are primarily available in English. For example, Spanish-speaking patients had over 10 times higher odds of needing help logging onto telehealth than English speakers.^26^ These challenges are often compounded by limited digital literacy: many LEP patients, especially older adults, may have lower comfort and familiarity with the digital technologies needed for telehealth. This digital literacy barrier intersects with linguistic challenges.^27^ While interpreter services could theoretically bridge communication gaps, interpreters frequently encounter challenges related to audio quality, communication flow, and access to technology during telehealth encounters.^28^ Furthermore, preference patterns appear to differ by language group, with Hispanic and Asian/Pacific Islander patients exhibiting lower interest in future telehealth utilization compared with White patients,^29^ suggesting potentially stronger preferences for in-person care among certain LEP populations.

### Decomposition analyses in health care research

The Blinder–Oaxaca decomposition method, initially developed within labor economics to analyze wage differentials,^30,31^ has emerged as a useful analytical tool for health disparity research. This regression-based approach systematically partitions outcome differences between groups into two components: an *explained* portion attributable to measurable between-group differences in observable characteristics, and an *unexplained* residual stemming from coefficient differences or unobserved factors.^32^ In health care research, this technique enables researchers to quantify how much of the disparity in outcomes or utilization is due to measurable socioeconomic and demographic factors, and how much remains unexplained, potentially reflecting structural barriers or systemic inequities.^32,33^ The method’s application in health care contexts has provided evidence on the mechanisms underlying persistent disparities. For instance, when applied to examine health care utilization gaps between Latino and White children in the United States, the Blinder–Oaxaca decomposition reveals not only how differences in socioeconomic factors and insurance status contribute to observed disparities but also quantifies the portion attributable to differential “returns” on these characteristics or other systemic factors not captured by covariates specified in the model.^33^

### The present study

Despite significant advances in telehealth research, several critical knowledge gaps persist. First, previous studies on racial/ethnic and linguistic disparities have often lacked granular analysis across diverse health care contexts. While overall telehealth adoption patterns have been well-documented, preferences for virtual versus in-person modalities likely vary substantially by specialty and visit purpose,^34,35^ ranging from mental health services to dermatology^36^ and primary care.^37,38^ Second, although studies have identified broad telehealth utilization patterns across various language groups,^20,23,34,35^ further investigation is needed to understand how these patterns manifest across specific visit types and levels of English proficiency. This intersection of visit type and language barriers remains understudied, particularly for Spanish- and Asian- language speakers.

Furthermore, the existing literature shows some methodological limitations. Previous studies have mainly relied on regression analyses to identify broad disparities,^20,23,34,35,39^ decomposition methods offer greater ability to disentangle and quantify the relative contributions of various factors to observed disparities in telehealth use. Although several studies have documented disparities in telehealth utilization,^16,23,40^ decomposition methods can clarify the extent to which observed disparities are attributable to measurable versus unmeasurable factors.^41^

The present study addresses these limitations by employing the nonlinear Blinder–Oaxaca decomposition method to investigate disparities in telehealth utilization across race/ethnicity, nativity, and language. By stratifying our analysis by visit purpose, we provide a more comprehensive understanding of how telehealth services are utilized across diverse health care needs and population subgroups. This approach enables a more precise examination of the factors underlying observed disparities, potentially informing more targeted interventions to enhance telehealth equity.

## Methods

### Data

We used CHIS data from 2021 and 2022, a period marked by the expansion of telehealth policies in California during the COVID-19 pandemic. Since the CHIS began incorporating telehealth-related questions in 2021, we aggregated our sample using datasets from the 2021 and 2022 survey rounds. The CHIS has been a continuous data source since 2007, collected through random-digit dialing of landlines and cell phones, targeting the noninstitutionalized population in California aged 18 to 64 years. Our dataset encompassed responses from 45,893 adults, collected in English, Spanish, and other languages.

### Variables

#### Telehealth service use

Our outcome variable, telehealth service use, was coded as binary variables for the following visit purposes: (a) mental health (mental/emotional problems), (b) primary care (check-ups, general questions in health, prescriptions, tests, results, follow-up), (c) acute care (flu, cold, allergies, infections, not feeling well, and other health problems), (d) chronic care (disease care and management), (e) orthopedics (arthritis, joint, back, muscle problems), and (6) dermatology and ophthalmology (skin and eyes).

#### LEP

LEP is defined as a person identified by the U.S. Census Bureau as being without the English proficiency of a “proficient” individual. Participants who reported that they spoke English “not at all,” “not well,” or “well” were grouped as LEP people, whereas non-LEP participants replied that they spoke English “very well.”

#### Explanatory variables

The explanatory variables include age group, gender, level of education, household income level, metropolitan or non-metropolitan area, type of insurance, and frequency of internet use.

Supplementary Appendix Table A1 shows that 53.5% of the non-LEP group and 43.9% of the LEP group received care from a doctor via video or telephone conversation instead of an in-person visit, indicating a significant 9.6% point difference in telehealth use between the two groups. The proportion of non-LEP respondents who used telehealth for mental health services was 15.8%, compared with 11.0% for those with LEP. In areas such as chronic care and orthopedics, the non-LEP group also showed higher usage rates than their LEP counterparts. On the other hand, the LEP group used telehealth more for acute care than the non-LEP group.

### Statistical analysis

We present descriptive statistics of variables, along with a comparison of means for individuals with and without LEP, in Supplementary Appendix Table A1. *T*-tests were employed to identify differences across these groups. The Blinder–Oaxaca decomposition method enabled us to dissect health care disparities between LEP and non-LEP individuals into components attributed to observed characteristics and unobserved heterogeneity, a technique previously applied to study racial and ethnic disparities in health insurance coverage and health care access and usage.^42,43^ This approach clarifies how disparities stem from differences in observed variables between groups and unobserved heterogeneity. To examine whether language-related disparities exist within ethnic groups, we conducted separate Oaxaca decompositions for the Hispanic/Latino and Asian subsamples based on individuals’ English proficiency. Given our binary outcome measures, we used nonlinear decomposition methods. We conducted statistical analyses using Stata.

## Results

### Evidence of linguistic barrier in telehealth usage: Decomposition by racial groups

Table 1 sets non-Hispanic Whites as the reference racial group and illustrates distinct racial disparities in the use of telehealth services by race and ethnicity. Based on CHIS data for 2021–2022, our findings indicate that African Americans used telehealth services more than non-Hispanic Whites by 4.1%. Conversely, both Hispanic and Asian populations showed a lower engagement with telehealth services, at 5.9% points and 11.3% points less than their non-Hispanic White counterparts, respectively.

**Table 1.** Telehealth Use Gap Decomposition

	vs. African American (1)	vs. Hispanic and Latino (2)	vs. Asian (3)
Baseline group: Non-Hispanic White	0.546^***^ (0.003)	0.546^***^ (0.003)	0.546^***^ (0.003)
Comparison group			
African American	0.586^***^ (0.011)	—	—
Hispanic and Latino	—	0.486^***^ (0.005)	—
Asian	—	—	0.433^***^ (0.006)
Difference	−0.041^***^ (0.011)	0.059^***^ (0.006)	0.113^***^ (0.007)
Explained	−0.007^**^ (0.003)	0.052^***^ (0.003)	0.008^***^ (0.002)
Unexplained	−0.034^***^ (0.011)	0.008 (0.006)	0.105^***^ (0.007)
Observations	24,833	34,470	29,919

Each column presents a separate regression for decomposition analysis. All specifications include respondents’ age group, gender, education, annual household income before taxes in the previous year, homeownership status, urbanity of residential address, insurance coverage status, frequency of internet usage, and survey year as regressors, although the table reports coefficients only for decomposition. Robust standard errors are reported in parentheses.

^**^ *p* < 0.05.

^***^ *p* < 0.01.

Blinder–Oaxaca decomposition helps explain differences between groups by separating them into explained and unexplained factors. The explained portion is attributed to differences in measurable covariates, such as age, gender, education, income, and insurance status, which are included in the model. In contrast, the unexplained portion is often interpreted as the effect of unmeasured factors, which could consist of discrimination, cultural differences, or—in this case—language barriers. Notably, a broad range of covariates was included but failed to account for approximately 14%^1^ of the observed discrepancy in the Hispanic group, and a substantial 92% of the gap in the Asian group. The results suggest that unobserved factors, including language skills, may influence the observed disparities, motivating our following analyses.

### Telehealth usage decomposition by language

#### LEP

Table 2 examines the disparity in telehealth usage by English proficiency, building on the evidence shown in Table 1. We divided our sample by LEP status.^2^ The pooled sample in column 1 of Table 2 compares the use of telehealth services by non-LEP and LEP individuals, regardless of the purpose of their visit. Non-LEP respondents used telehealth at a rate of 53.7%, compared with a lower rate of 43.9% for LEP respondents. Approximately 38% of the difference remained unexplained by the demographic and socioeconomic covariates. In contrast, we found higher use of telehealth services in primary and acute care among LEP patients than non-LEP patients.

**Table 2.** Telehealth Utilization Disparities by Proficiency in English and Health Concerns

	Pooled (1)	Mental health (2)	Primary care (3)	Acute care (4)	Chronic care (5)	Orthopedics (6)	Dermatology/ophthalmology (7)
Baseline group: Non-LEP	0.535^***^ (0.003)	0.158^***^ (0.003)	0.481^***^ (0.004)	0.253^***^ (0.003)	0.176^***^ (0.003)	0.189^***^ (0.003)	0.157^***^ (0.003)
Comparison group: LEP	0.439^***^ (0.005)	0.109^***^ (0.005)	0.500^***^ (0.007)	0.287^***^ (0.007)	0.151^***^ (0.005)	0.176^***^ (0.006)	0.149^***^ (0.005)
Difference	0.096^***^ (0.006)	0.049^***^ (0.005)	−0.018^**^ (0.008)	−0.034^***^ (0.007)	0.025^***^ (0.006)	0.0132^**^ (0.006)	0.008 (0.006)
Explained	0.059^***^ (0.004)	−0.010^**^ (0.005)	−0.001 (0.006)	−0.019^***^ (0.005)	0.019^***^ (0.005)	0.008 (0.005)	0.002 (0.004)
Unexplained	0.036^***^ (0.007)	0.059^***^ (0.007)	−0.017 (0.010)	−0.015* (0.009)	0.006 (0.007)	0.005 (0.008)	0.006 (0.007)
Observations	23,556	23,556	23,556	23,556	23,556	23,556	23,556

Robust standard errors are reported in parentheses.

LEP refers to respondents who reported their English-speaking ability as “not at all,” “not well,” or just “well,” whereas the non-LEP group comprises individuals who indicated that they spoke English “very well.” The type of visit categories are binary indicators, so multiplying the coefficients by 100 allows for interpretation as percentages. (1): The dependent variable is whether telehealth was used. (2)–(7): The samples used are conditional on the use of telehealth services, and survey participants were allowed to select all that applied.

^*^ *p* < 0.1.

^**^ *p* < 0.05.

^***^ *p* < 0.01.

Looking into specific visit purposes, the disparity was most apparent within mental health care (column 2 in Table 2), where LEP users accessed services 4.87% points less frequently than their non-LEP counterparts. This represented the largest gap among the six types of visits: mental health, primary care, acute care, chronic care, orthopedic care, and dermatology/ophthalmology. More importantly, the entire disparity was not explained by covariates in the model. The disparity in telehealth use among LEP patients compared with non-LEP patients was also observed in chronic and orthopedic care, albeit to a lesser extent.

**FIG. 1.**
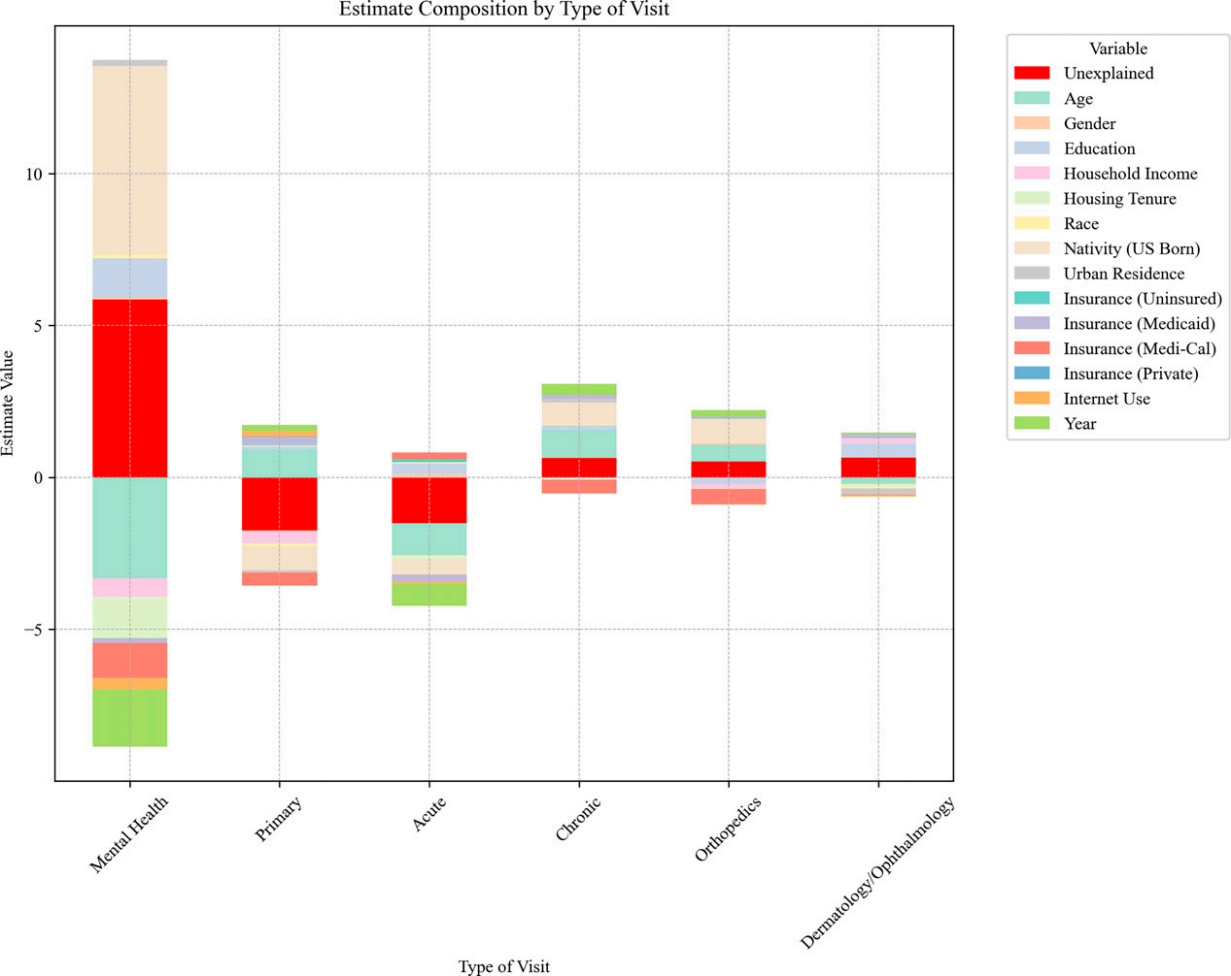
Telehealth use gap by proficiency in English and health concerns. Each bar represents the total telehealth utilization gap between limited English proficient (LEP) and English proficient respondents across health care service categories, with each segment representing the contribution of a specific covariate. For visit types with positive overall gaps (where non-LEP usage exceeds LEP usage), segments above zero indicate variables that widen the gap, while negative coefficients indicate factors that narrow the gap. Conversely, for negative gaps (where LEP usage exceeds non-LEP usage), the interpretation is reversed. For detailed information on each covariate, please refer to Supplementary Appendix Table A1.

Figure 1 illustrates the decomposed telehealth utilization gaps between LEP and non-LEP respondents across the six health care service categories. Mental health services exhibit the largest disparity, primarily driven by unexplained factors. Positive coefficients in the unexplained portion indicate widening disparities that cannot be attributed to observed sociodemographic characteristics, suggesting potential linguistic or cultural barriers. Conversely, the negative gaps observed in primary and acute care suggest that LEP patients actually utilize these telehealth services more frequently than their non-LEP counterparts. These differences are partially explained by observable demographic factors and partially attributed to unexplained factors, which could include cultural preferences, health care-seeking behavior, or unmeasured access barriers.

#### Home language

Based on our subsamples for the language spoken at home (columns 2, 3, and 4 in Table 3), we found the telehealth use gap persisted, regardless of whether respondents spoke Spanish, an Asian language, or a language other than English at home. The rate of telehealth use among other language speakers was higher, regardless of their LEP status, compared with Spanish or Asian language speakers; however, LEP patients still used telehealth services less frequently than their non-LEP counterparts within the other language speaker group by 7.3% points (see Supplementary Appendix Table A3 for detailed utilization rates by language group and visit type). Approximately 44.9% of LEP patients who were Spanish speakers at home used telehealth services, whereas only 39.5% of LEP patients spoke Asian languages at home. Although not statistically significant, the negative sign of the explained component suggests that observable covariates do not substantially contribute to explaining the gap, further indicating that the disparity likely originates from unobserved factors. The entire telehealth use gap for Asian language speakers was not explained by the covariates included, and the estimates were statistically significant despite the small sample size. Table A2 in the Supplementary Appendix also examines the gap by the six purposes of visit. A large portion of the telehealth use gap for mental health concerns remained unexplained among all the home language groups.

**Table 3.** Telehealth Use Disparities by Proficiency in English and Language at Home

	Pooled (1)	Spanish speakers only (2)	Asian language speakers only (3)	Other language speakers only (4)
Baseline group: Non-LEP	0.535^***^ (0.003)	0.492^***^ (0.009)	0.468^***^ (0.014)	0.566^***^ (0.015)
Comparison group: LEP	0.439^***^ (0.005)	0.449^***^ (0.007)	0.395^***^ (0.009)	0.493^***^ (0.013)
Difference	0.096^***^ (0.006)	0.043^***^ (0.012)	0.073^***^ (0.017)	0.073^***^ (0.019)
Explained	0.059^***^ (0.004)	0.025^**^ (0.011)	−0.005 (0.013)	−0.033 (0.025)
Unexplained	0.036^***^ (0.007)	0.018 (0.015)	0.078^***^ (0.021)	0.106^***^ (0.031)
Observations	45,893	8,295	4,411	2,753

Asian language refers to Chinese, Vietnamese, Korean, or other Asian languages. This category includes both monolingual speakers of these languages and bilingual speakers who use English alongside an Asian language. The Spanish category encompasses monolingual Spanish speakers as well as bilingual English-Spanish speakers. The other languages category includes monolingual speakers of languages not listed above (non-English, non-Spanish, non-Chinese, non-Vietnamese, non-Korean), bilingual speakers of English with a European language, multilingual speakers of two or more languages outside the previously specified Asian and Spanish categories, and bilingual speakers of English with any language other than Spanish, Chinese, Vietnamese, or Korean.

^**^ *p* < 0.05

^***^ *p* < 0.01

## Discussion

### Primary findings

Our study provides compelling evidence of linguistic barriers in telehealth utilization during the COVID-19 pandemic. We identified significant disparities in telehealth use between non-Hispanic Whites and other racial and ethnic groups, with particularly pronounced differences among Hispanic and Asian populations. The decomposition analysis revealed that a substantial portion of these disparities remains unexplained by conventional socioeconomic determinants and internet use patterns, suggesting the influence of unmeasured factors.

These unexplained disparities were particularly pronounced among Asian and other non-Spanish language speakers, indicating a critical need for further research focusing on speakers of less commonly encountered languages in health care settings. The extraordinary linguistic diversity within Asian communities presents unique challenges for telehealth implementation, encompassing more than 100 distinct languages and dialects.^44,45^ While language-specific telehealth infrastructure is essential for equitable access, the relatively small population sizes of individual Asian language communities make such implementations resource-intensive and logistically complex. Beyond linguistic considerations, cultural expectations significantly influence health care delivery preferences among Asian immigrant communities. Research indicates that Asian American patients often express preferences for providers who share their linguistic and ethnic background, perceiving such providers as possessing greater cultural understanding and sensitivity.^46^ These cultural factors, alongside linguistic barriers, likely contribute to the substantial unexplained component of observed disparities in telehealth utilization.

We found that the disparity in telehealth use between LEP and non-LEP patients was most pronounced in mental health care, likely reflects the nature of mental health services heavily relying on both verbal and non-verbal communication to build therapeutic reliance.^47–49^ The effectiveness of mental health treatment often depends on the patient’s ability to express their thoughts, feelings, and experiences.^50,51^ When using telehealth platforms, this communication is conducted through voice or video calls, which can be particularly challenging for clinicians to establish rapport and assess non-verbal signs of mental illness symptoms.^52^ The reliance on language to convey emotional states means that any barrier to communication can significantly impact the quality of care,^53^ resulting in telehealth care being less accessible to LEP patients.

The lack of language-concordant providers^54^ and insufficient interpreter support^55^ on telehealth platforms may further exacerbate the language barrier. The behavioral health workforce shortage is particularly acute for language-concordant care, with Hispanic/Latino providers comprising only 6.6% of psychiatrists^56^ and 7.95% of psychologists^57^ despite representing 19% of the U.S. population. Although integrating professional interpreters^58^ via videoconferencing could offer a solution,^59^ implementation challenges include impediments to therapeutic rapport in mental health care.^60^ These limitations make the presence of language-concordant mental health providers particularly critical for delivering culturally competent telemental health services to LEP populations.^61^

In contrast, we found higher telehealth use among LEP patients for primary and acute care services compared to non-LEP patients, aligning with a recent study.^38^ One possible explanation is that LEP patients may have stronger trust and continuity in care^62^ with their primary care clinicians, making telehealth an acceptable option compared with specialty services. LEP communities often harbor fears of misunderstanding or mistreatment in health care (due to past discrimination or communication difficulties),^63^ so a trusted provider relationship is critical before they feel comfortable transitioning that relationship online. In addition, primary care frequently involves general health inquiries, prescription management, and follow-ups that may not necessitate extensive medical vocabulary or conversation.^64^ Acute care for conditions such as flu or minor infections often involves more straightforward communication and allows providers to rely on visual cues.^65^ On the other hand, LEP individuals are significantly underrepresented in outpatient specialties; for example, they have much lower visit rates in subspecialties and surgery compared with primary care.^66^ This suggests that communication barriers, such as the need for specialized vocabulary and coordinated interpretation, pose greater obstacles to specialty services more than general primary care.

### Policy implications

Our findings have implications for health policy and practice. First, the unexplained disparities in telehealth use highlight the barriers to health care delivery, particularly for patients with LEP.^20,23,34,35,39^ This disparity was most pronounced in telemental health services, where effective communication is fundamental to care delivery.^67^ Second, addressing these disparities requires systematic efforts at multiple levels: (1) development and implementation of language-concordant telehealth services,^54^ (2) provision of professional interpreters and translated materials,^51,68^ and (3) enhanced provider training in cultural competency.^69^ Finally, previous studies suggest that telehealth can be an effective modality for engaging LEP populations only when designed with specific features in mind, including multilingual user interfaces,^70^ seamless scheduling logistics for interpretation,^36^ and building provider–patient trust through cultural competency.^71,72^

## Conclusions

This study has several limitations that warrant consideration. First, our reliance on self-reported data from the CHIS introduces potential recall bias. Second, despite controlling for key demographic and socioeconomic factors, our analysis could not account for several potentially significant variables, including attitudes toward telehealth,^73^ digital literacy levels,^74,75^ provider–patient language concordance,^76^ and family involvement in medical decisions.^77^ Future research would benefit from mixed-methods studies that complement quantitative analysis with qualitative interviews to gain a deeper understanding of the cultural factors influencing telehealth use. Third, our study’s focus on California limits its generalizability to states with different demographic compositions and health care systems.

Despite these limitations, our findings provide valuable evidence on the impact of linguistic barriers on telehealth utilization across diverse health care services, with particularly pronounced disparities in access to mental health care. These results have important implications for health care policy and practice. Future research should evaluate the effectiveness of language-concordant telehealth interventions,^72^ including examining how different approaches to providing interpreter services,^28^ translated materials,^78^ and culturally adapted telehealth platforms.

In addition, longitudinal studies are needed to examine how linguistic barriers in telehealth access affect health outcomes and health care expenditures, particularly within mental health services, where we observed the most substantial disparities. Understanding these relationships will become increasingly critical as health care systems continue to expand telehealth offerings and work to ensure equitable access across linguistically diverse communities. These findings underscore the need for systematic efforts to develop and implement linguistically appropriate telehealth services, thereby preventing the exacerbation of existing health care disparities in an increasingly digital health care environment.

## Supplementary Material

Supplementary Appendix

## Data Availability

The California Health Interview Survey dataset analyzed during the current study is publicly available in the UCLA Center for Health Policy Research (CHIS), https://healthpolicy.ucla.edu/.

## Notes

^1^
0.008/0.059, 0.105/0.113

^2^
43.9% of the sample respondents were with LEP.

## Abbreviations Used

CHIS: California Health Interview Survey
IRB: Institutional Review Board
LEP: Limited English proficiency
NIH: National Institutes of Health
UCLA: University of California, Los Angeles
