# Supplementary material for: Explaining Racial/Ethnic Disparities in Telehealth Use with Different Levels of English Proficiency: A Decomposition Approach
Source: Telemed Rep. 2025 Sep 29;6(1):268–78. doi: 10.1177/26924366251379188 (PMC12549173; doi:10.1177/26924366251379188)
Supplement: Supplementary Appendix [file 26924366251379188_supplementary_appendix.docx]

**Appendix**

Appendix Table A1: Telehealth Utilization and Sample Characteristics by Limited English Proficiency (LEP)

|  | Pooled  (1) | Non-LEP  (2) | | LEP  (3) | | Difference  (4) | |  |
| --- | --- | --- | --- | --- | --- | --- | --- | --- |
|  | Mean | Mean | SD | Mean | SD | (2)-(3) | *p*-value |  |
| **Telehealth Use** |  |  |  |  |  |  |  |  |
| Yes | 0.513 | 0.535 | (0.499) | 0.439 | (0.496) | 0.096 | [<0.000] |  |
| Mental Health | 0.149 | 0.158 | (0.365) | 0.110 | (0.312) | 0.048 | [<0.000] |  |
| Primary Care | 0.485 | 0.481 | (0.500) | 0.500 | (0.500) | -0.019 | [0.024] |  |
| Acute Care | 0.260 | 0.253 | (0.435) | 0.287 | (0.452) | -0.034 | [<0.000] |  |
| Chronic Care | 0.171 | 0.176 | (0.381) | 0.151 | (0.358) | 0.025 | [<0.000] |  |
| Orthopedics | 0.186 | 0.189 | (0.391) | 0.175 | (0.380) | 0.013 | [0.036] |  |
| Dermatology/Ophthalmology | 0.156 | 0.157 | (0.364) | 0.149 | (0.356) | 0.008 | [0.167] |  |
| No | 0.487 | 0.465 | (0.499) | 0.561 | (0.496) | -0.096 | [<0.000] |  |
| **Race/Ethnicity** |  |  |  |  |  |  |  |  |
| Hispanic and Latino | 0.256 | 0.179 | (0.383) | 0.523 | (0.499) | -0.345 | [<0.000] |  |
| Non-Hispanic white | 0.495 | 0.609 | (0.488) | 0.102 | (0.302) | 0.507 | [<0.000] |  |
| African American | 0.046 | 0.057 | (0.231) | 0.009 | (0.093) | 0.048 | [<0.000] |  |
| American Indian/Alaskan Native | 0.006 | 0.008 | (0.088) | 0.001 | (0.036) | 0.007 | [<0.000] |  |
| Asian | 0.157 | 0.100 | (0.300) | 0.354 | (0.478) | -0.254 | [<0.000] |  |
| Other | 0.040 | 0.048 | (0.214) | 0.011 | (0.106) | 0.037 | [<0.000] |  |
| **Gender** |  |  |  |  |  |  |  |  |
| Female | 0.565 | 0.570 | (0.495) | 0.548 | (0.498) | 0.022 | [<0.000] |  |
| Male | 0.435 | 0.430 | (0.495) | 0.452 | (0.498) | -0.022 | [<0.000] |  |
| **Age** |  |  |  |  |  |  |  |  |
| 18-29 | 0.094 | 0.088 | (0.283) | 0.116 | (0.320) | -0.028 | [<0.000] |  |
| 30-39 | 0.137 | 0.131 | (0.338) | 0.157 | (0.364) | -0.026 | [<0.000] |  |
| 40-49 | 0.153 | 0.142 | (0.349) | 0.190 | (0.392) | -0.047 | [<0.000] |  |
| 50-64 | 0.316 | 0.316 | (0.465) | 0.316 | (0.465) | <0.000 | [0.942] |  |
| 65+ | 0.300 | 0.322 | (0.467) | 0.221 | (0.415) | 0.101 | [<0.000] |  |
| **Education** |  |  |  |  |  |  |  |  |
| Less than high school | 0.048 | 0.023 | (0.149) | 0.137 | (0.343) | -0.114 | [<0.000] |  |
| High school | 0.122 | 0.112 | (0.316) | 0.155 | (0.362) | -0.043 | [<0.000] |  |
| Some college | 0.278 | 0.287 | (0.452) | 0.244 | (0.430) | 0.043 | [<0.000] |  |
| College or above | 0.552 | 0.578 | (0.494) | 0.464 | (0.499) | 0.114 | [<0.000] |  |
| **Household income** |  |  |  |  |  |  |  |  |
| 0-49,999 | 0.319 | 0.289 | (0.453) | 0.422 | (0.494) | -0.133 | [<0.000] |  |
| 50,000-99,999 | 0.270 | 0.273 | (0.445) | 0.263 | (0.440) | 0.010 | [0.041] |  |
| 100,000-149,999 | 0.170 | 0.180 | (0.384) | 0.136 | (0.343) | 0.044 | [<0.000] |  |
| 150,000- | 0.240 | 0.258 | (0.438) | 0.179 | (0.383) | 0.079 | [<0.000] |  |
| **Tenure** |  |  |  |  |  |  |  |  |
| Own | 0.636 | 0.663 | (0.473) | 0.544 | (0.498) | 0.119 | [<0.000] |  |
| Rent | 0.364 | 0.337 | (0.473) | 0.456 | (0.498) | -0.119 | [<0.000] |  |
| **Born in US** |  |  |  |  |  |  |  |  |
| Yes | 0.740 | 0.866 | (0.340) | 0.304 | (0.460) | 0.562 | [<0.000] |  |
| No | 0.260 | 0.134 | (0.340) | 0.696 | (0.460) | -0.562 | [<0.000] |  |
| **Metropolitan** |  |  |  |  |  |  |  |  |
| Yes | 0.924 | 0.909 | (0.288) | 0.977 | (0.149) | -0.068 | [<0.000] |  |
| No | 0.076 | 0.091 | (0.288) | 0.023 | (0.149) | 0.068 | [<0.000] |  |
| **Insurance type** |  |  |  |  |  |  |  |  |
| Uninsured | 0.040 | 0.029 | (0.167) | 0.080 | (0.271) | -0.051 | [<0.000] |  |
| Medicare | 0.300 | 0.323 | (0.468) | 0.219 | (0.414) | 0.104 | [<0.000] |  |
| Medi-Cal | 0.172 | 0.142 | (0.349) | 0.279 | (0.448) | -0.137 | [<0.000] |  |
| Other plans purchased on their own | 0.057 | 0.056 | (0.230) | 0.061 | (0.239) | -0.005 | [0.053] |  |
| **Internet use** |  |  |  |  |  |  |  |  |
| Less than a few times a day | 0.157 | 0.127 | (0.333) | 0.258 | (0.438) | -0.131 | [<0.000] |  |
| A few times a day | 0.313 | 0.305 | (0.461) | 0.341 | (0.474) | -0.036 | [<0.000] |  |
| Many times a day | 0.337 | 0.359 | (0.480) | 0.258 | (0.438) | 0.101 | [<0.000] |  |
| Almost constantly | 0.193 | 0.207 | (0.406) | 0.141 | (0.349) | 0.066 | [<0.000] |  |
| **Survey year** |  |  |  |  |  |  |  |  |
| 2021 | 0.533 | 0.596 | (0.491) | 0.312 | (0.463) | 0.284 | [<0.000] |  |
| 2022 | 0.467 | 0.404 | (0.491) | 0.688 | (0.463) | -0.284 | [<0.000] |  |

Notes: Column (1) shows the average value of each indicator for the entire sample, regardless of LEP status, while columns (2) and (3) show for survey respondents who did not have limited English proficiency (Non-LEP) and who had limited English proficiency (LEP), respectively, with standard deviations reported in parentheses. Column (4) presents the difference in means between the Non-LEP (column (2)) and LEP (column (3)) groups, with the *p*-values brackets to indicate if the observed difference is statistically significant. Survey respondents could report whether they used telehealth services (Yes) or not (No), the two of which totals 1 (100%). Conditional on the response is “Yes,” there are six visit purposes (Mental Health, Primary Care, Acute Care, Chronic Care, Orthopedics, Dermatology/Ophthalmology). The respondents could indicate what their telehealth visit was for. All variables except for the survey years are self-reported. The table reports only five age groups and four household income bins for space; however, all specifications control for the age and household income variables in original categories: 14 categories for age and 19 categories for household income.

**Appendix Table A2**: Telehealth Utilization by Purpose of Visit, Subgroup by Home Languages

|  | Pooled | Spanish speakers only | Asian language speakers only | Other language speakers only |  | Pooled | Spanish speakers only | Asian language speakers only | Other language speakers only |
| --- | --- | --- | --- | --- | --- | --- | --- | --- | --- |
|  | (1a) | (2a) | (3a) | (4a) |  | (1b) | (2b) | (3b) | (4b) |
| ***Panel A. Mental Health*** | | | | | ***Panel B. Primary Care*** | | | | |
| Baseline group: Not LEP | 0.158*** | 0.153*** | 0.114*** | 0.168*** | Baseline group: Not LEP | 0.481*** | 0.474*** | 0.484*** | 0.506*** |
|  | (0.00264) | (0.00969) | (0.0133) | (0.0146) |  | (0.00362) | (0.0134) | (0.0209) | (0.0196) |
| Comparison group: LEP | 0.109*** | 0.120*** | 0.0706*** | 0.137*** | Comparison group: LEP | 0.500*** | 0.484*** | 0.538*** | 0.487*** |
|  | (0.00464) | (0.00655) | (0.00722) | (0.0123) |  | (0.00744) | (0.0101) | (0.0140) | (0.0178) |
| Difference | 0.0487*** | 0.0329** | 0.0434** | 0.0311 | Difference | -0.0185* | -0.00987 | -0.0539* | 0.0188 |
|  | (0.00534) | (0.0117) | (0.0151) | (0.0191) |  | (0.00828) | (0.0168) | (0.0252) | (0.0265) |
| Explained | -0.00932* | 0.00254 | 0.0110 | -0.0386 | Explained | -0.00106 | -0.0166 | 0.00562 | -0.0746 |
|  | (0.00453) | (0.0107) | (0.0113) | (0.0243) |  | (0.00624) | (0.0175) | (0.0214) | (0.0388) |
| Unexplained | 0.0580*** | 0.0303* | 0.0324 | 0.0697* | Unexplained | -0.0174 | 0.00673 | -0.0595 | 0.0934* |
|  | (0.00700) | (0.0145) | (0.0167) | (0.0281) |  | (0.0102) | (0.0240) | (0.0321) | (0.0468) |
| Observations | 23556 | 3846 | 1830 | 1442 | Observations | 23556 | 3846 | 1830 | 1442 |
| ***Panel C. Acute Care*** | | | | | ***Panel D. Chronic Care*** | | | | |
| Baseline group: Not LEP | 0.253*** | 0.309*** | 0.272*** | 0.274*** | Baseline group: Not LEP | 0.176*** | 0.146*** | 0.146*** | 0.159*** |
|  | (0.00315) | (0.0124) | (0.0186) | (0.0174) |  | (0.00276) | (0.00950) | (0.0148) | (0.0143) |
| Comparison group: LEP | 0.287*** | 0.302*** | 0.249*** | 0.303*** | Comparison group: LEP | 0.151*** | 0.153*** | 0.149*** | 0.146*** |
|  | (0.00673) | (0.00924) | (0.0122) | (0.0164) |  | (0.00532) | (0.00725) | (0.0100) | (0.0126) |
| Difference | -0.0341*** | 0.00699 | 0.0227 | -0.0296 | Difference | 0.0254*** | -0.00723 | -0.00359 | 0.0131 |
|  | (0.00743) | (0.0155) | (0.0223) | (0.0239) |  | (0.00600) | (0.0119) | (0.0179) | (0.0191) |
| Explained | -0.0178** | 0.00368 | 0.0108 | -0.0482 | Explained | 0.0196*** | -0.0220 | -0.00670 | -0.0518* |
|  | (0.00551) | (0.0155) | (0.0187) | (0.0346) |  | (0.00464) | (0.0132) | (0.0160) | (0.0259) |
| Unexplained | -0.0164 | 0.00331 | 0.0120 | 0.0186 | Unexplained | 0.00579 | 0.0148 | 0.00311 | 0.0649* |
|  | (0.00918) | (0.0209) | (0.0275) | (0.0410) |  | (0.00737) | (0.0183) | (0.0241) | (0.0311) |
| Observations | 23556 | 3846 | 1830 | 1442 | Observations | 23556 | 3846 | 1830 | 1442 |
| ***Panel E. Orthopedics*** | | | | | ***Panel F. Dermatology/Ophthalmology*** | | | | |
| Baseline group: Not LEP | 0.189*** | 0.165*** | 0.172*** | 0.190*** | Baseline group: Not LEP | 0.157*** | 0.154*** | 0.198*** | 0.190*** |
|  | (0.00284) | (0.01000) | (0.0158) | (0.0153) |  | (0.00264) | (0.00973) | (0.0167) | (0.0153) |
| Comparison group: LEP | 0.176*** | 0.180*** | 0.156*** | 0.193*** | Comparison group: LEP | 0.149*** | 0.133*** | 0.167*** | 0.170*** |
|  | (0.00566) | (0.00775) | (0.0102) | (0.0141) |  | (0.00530) | (0.00684) | (0.0105) | (0.0134) |
| Difference | 0.0132* | -0.0152 | 0.0164 | -0.00329 | Difference | 0.00834 | 0.0213 | 0.0308 | 0.0196 |
|  | (0.00633) | (0.0126) | (0.0188) | (0.0208) |  | (0.00592) | (0.0119) | (0.0197) | (0.0204) |
| Explained | 0.00770 | 0.00811 | -0.0345* | -0.0392 | Explained | 0.00190 | -0.000372 | 0.0164 | -0.00108 |
|  | (0.00476) | (0.0133) | (0.0162) | (0.0302) |  | (0.00447) | (0.0115) | (0.0165) | (0.0299) |
| Unexplained | 0.00547 | -0.0234 | 0.0508* | 0.0359 | Unexplained | 0.00643 | 0.0217 | 0.0144 | 0.0206 |
|  | (0.00786) | (0.0188) | (0.0249) | (0.0363) |  | (0.00737) | (0.0157) | (0.0250) | (0.0360) |
| Observations | 23556 | 3846 | 1830 | 1442 | Observations | 23556 | 3846 | 1830 | 1442 |

Notes: The dependent variables are whether telehealth was used for Mental Health visit (for Panel A), Primary Care visit (Panel B), Acute Care visit (Panel C), Chronic Care visit (Panel D), Orthopedics visit (Panel E), Dermatology/Ophthalmology visits (Panel F). Type of visit categories are binary indicators, so multiplying the coefficients by 100 allows interpretation as percentages. LEP refers to respondents who reported their English-speaking ability as ‘not at all,’ ‘not well,’ or just ‘well,’ while the non-LEP group comprises individuals who indicated that they speak English ‘very well.’ Columns (1a, b) show the decomposition results for the pooled sample, regardless of the respondents' home languages. Columns (2a, b), (3a, b), and (4a, b) show the decomposition results for the respondents who use Spanish, Asian languages, and other languages at home, respectively. Asian languages are Chinese, Vietnamese, Korean, English, Chinese, or English and other Asian languages. Spanish includes Spanish or English and Spanish. Other languages include another language that is not English, Spanish, Chinese, Vietnamese, or Korean, English and European language, other two languages or over that are not English, Spanish, Chinese, Vietnamese or Korean, or English and another language that is not English, Spanish, Chinese, Vietnamese, or Korean. The samples used are *conditional on* the usage of telehealth services, and survey participants were allowed to select all that apply. All variables except for the survey years are self-reported. Robust standard errors are reported in parentheses. *** *p*<0.01, ***p*<0.05, * *p*<0.1.

**Appendix Table A3**: Telehealth Utilization by Language at Home and Health Concerns

|  | **Mental Health** | **Primary** | **Acute** | **Chronic** | **Orthopedics** | **Dermatology/ Ophthalmology** | **Ever Visited** |
| --- | --- | --- | --- | --- | --- | --- | --- |
|  | **(1)** | **(2)** | **(3)** | **(4)** | **(5)** | **(6)** | **(7)** |
| English | 2,621 | 7,905 | 4,062 | 2,964 | 3,146 | 2,547 | 16,445 |
|  | (15.9%) | (48.1%) | (24.7%) | (18.0%) | (19.1%) | (15.5%) |  |
| Spanish | 507 | 1,848 | 1,170 | 579 | 673 | 542 | 3,847 |
|  | (13.2%) | (48.0%) | (30.4%) | (15.1%) | (17.5%) | (14.1%) |  |
| Asian | 156 | 956 | 469 | 271 | 294 | 324 | 1,833 |
|  | (8.5%) | (52.2%) | (25.6%) | (14.8%) | (16.0%) | (17.7%) |  |
| Other | 218 | 715 | 418 | 219 | 276 | 258 | 1,442 |
|  | (15.1%) | (49.6%) | (29.0%) | (15.2%) | (19.1%) | (17.9%) |  |

Notes: Asian language refers to Chinese, Vietnamese, Korean, or other Asian languages. This category includes both monolingual speakers of these languages and bilingual speakers who use English alongside an Asian language. The Spanish category encompasses monolingual Spanish speakers as well as bilingual English-Spanish speakers. The other languages category includes monolingual speakers of languages not listed above (non-English, non-Spanish, non-Chinese, non-Vietnamese, non-Korean), bilingual speakers of English with a European language, multilingual speakers of two or more languages outside the previously specified Asian and Spanish categories, and bilingual speakers of English with any language other than Spanish, Chinese, Vietnamese, or Korean. The last column (7), “Ever Visited,” counts the number of respondents who have ever utilized telehealth care. Accordingly, the sum of columns (1)-(7) may be greater than or equal to the count represented in column (7).
